# The role of the gut microbiota and fecal microbiota transplantation in neuroimmune diseases

**DOI:** 10.3389/fneur.2023.1108738

**Published:** 2023-02-01

**Authors:** Nan Wu, Xizhi Li, He Ma, Xue Zhang, Bin Liu, Yuan Wang, Qi Zheng, Xueli Fan

**Affiliations:** ^1^Department of Neurology, Binzhou Medical University Hospital, Binzhou, China; ^2^Institute for Metabolic and Neuropsychiatric Disorders, Binzhou Medical University Hospital, Binzhou, China

**Keywords:** gut microbiota, fecal microbiota transplantation (FMT), neuroimmune diseases, myasthenia gravis (MG), multiple sclerosis (MS), neuromyelitis optica spectrum disorders (NMOSDs), autoimmune encephalitis (AIE), Guillain–Barré syndrome (GBS)

## Abstract

The gut microbiota plays a key role in the function of the host immune system and neuroimmune diseases. Alterations in the composition of the gut microbiota can lead to pathology and altered formation of microbiota-derived components and metabolites. A series of neuroimmune diseases, such as myasthenia gravis (MG), multiple sclerosis (MS), neuromyelitis optica spectrum disorders (NMOSDs), Guillain–Barré syndrome (GBS), and autoimmune encephalitis (AIE), are associated with changes in the gut microbiota. Microecological therapy by improving the gut microbiota is expected to be an effective measure for treating and preventing some neuroimmune diseases. This article reviews the research progress related to the roles of gut microbiota and fecal microbiota transplantation (FMT) in neuroimmune diseases.

## 1. The gut microbiota

The gut is colonized by a multitude of microorganisms, commonly referred to as the gut microbiota. Although most of these organisms are bacteria, others include archaea, protists, fungi, and viruses. The gut microbiota comprises about 1,200 bacterial species. The dominant phyla in the intestinal tract belong mainly to *Bacteroidetes, Firmicutes, Actinobacteria, and Proteobacteria* (1, 2). The gut microbiota forms a long-term, dynamically balanced symbiotic relationship with the host that plays an important role in human immunity and metabolism. The gut microbiota affects the synthesis of polysaccharides, glycosides, vitamins, and essential amino acids. Additionally, the gut microbiota catalyzes the metabolism of drugs, carcinogens, and hormones and reduces the synthesis of histamine to play a detoxification role. Moreover, the gut microbiota performs protective functions by producing various antibacterial and bacteriostatic substances and degrading bacterial toxins (3, 4).

Gut microbiota dysbiosis can lead to pathology and altered formation of microbiota-derived components and metabolites, resulting in dysregulation of the immune system and metabolism (5).

## 2. Gut-brain axis (GBA)

The gut microbiota, as a well-known part of bidirectional neurohormonal communication system, affects gut pathophysiology and central nervous system (CNS) function through multiple bidirectional pathways of the GBA. The gut microbiota is transmitted to the brain *via* multiple afferent signaling pathways, including neural (vagal and spinal afferents), endocrine (cytokines, metabolites, and microbial signaling molecules), the hypothalamic–pituitary–adrenal (HPA) axis, and immune signaling (6, 7). Conversely, signals from CNS and neuroendocrine systems might monitor and regulate changes in the gut microbiota to adapt to environmental changes (8, 9).

### 2.1. Vagus nerve

The vagus nerve is the main conduit for communication between the brain and the gut. It connects the gastrointestinal tract with the nucleus tractus solitarius and the higher emotional regulation network (10). Numerous microbial-derived metabolites are able to impact vagal activation. Butyrate can directly activate vagal afferent terminals in the gut, and SCFA oleate activates the vagus nerve *via* the CCK-A receptor (11). In addition, toll-like receptor 4, a pattern recognition receptor for expression of vagus nerve fibers, can directly recognize bacterial products and thus activate the vagus nerve (12). Lactobacillus rhamnose-JB-1 alters the expression of γ-aminobutyric acid (GABA) receptors in the amygdala and hippocampus, and improves anxiety-like behaviors in mice (13). Most effects of L. rhamnosus JB-1 are lost in vagotomized mice, emphasize beneficial roles for the vagus nerve in microbiota-CNS communication (14). Futhermore, vagotomy can block central signaling of Lactobacillus and Bifidobacterium species, aggravate anxiety and depression related behavior in mice (13, 15). On the contrary, targeted vagal stimulation can suppress lipopolysaccharides (LPS)-induced proinflammatory cytokine production by microglia (16, 17). Interestingly, patients who underwent a full truncal vagotomy for treatment of peptic ulcer disease have a decreased risk of certain neurological diseases such as Parkinson's disease when they enter old age (18).

### 2.2. Microbe-derived metabolites (short-chain fatty acids, SCFAs)

**Figure 1:**
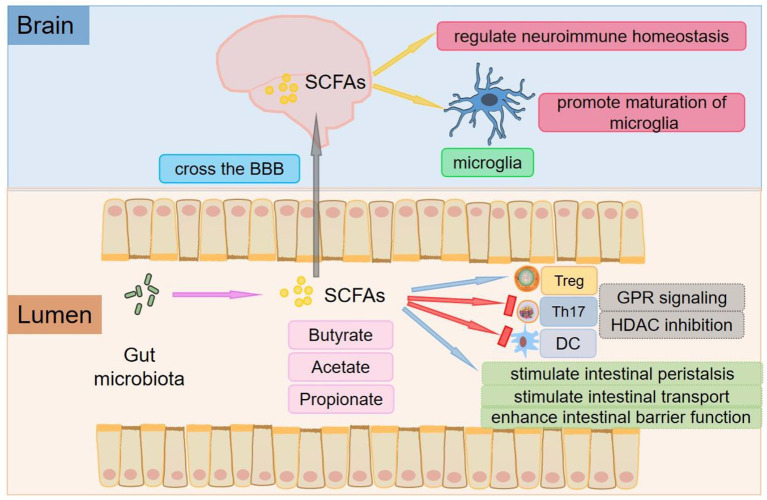
The roles of SCFAs. The main SCFAs are acetate, propionate, and butyrate, which are produced in the process of microbial fermentation of indigestible dietary fibers or resistant starch. SCFAs are key mediators of metabolism and immune cell function in the gut mucosa and can stimulate intestinal peristalsis and intestinal transport and affect the integrity of the intestinal barrier. SCFAs exert anti-inflammatory effects beyond the gut, which help increase the number of Tregs and inhibit the differentiation of Th17 cells. SCFAs can promote the differentiation of Tregs by inhibiting HDAC or activating GPR109A/HCA2. Tregs can inhibit the function of antigen-presenting cells and other effector T cells and suppress immune responses. SCFAs also inhibit DCs development and regulate antigen presentation of DCs by inhibiting HDAC and interacting with FFARs. SCFAs can not only cross the BBB and control neuroimmune homeostasis but also play an important role in the recovery and maturation of microglial function.

Some studies have found that direct contact between microbiota-derived components and metabolites (vitamins, bile acids, LPS, SCFAs, derivatives of tryptophan metabolism, etc.) and host metabolite sensing receptors expressed in immune cells of the intestinal mucosa initiates the mucosal immune response and activates macrophages, dendritic cells (DCs), and T cells. The gut microbiota is indispensable for host immune functions and actively regulates immune homeostasis in the peripheral and CNS (19). SCFAs are the most widely studied metabolites involved in regulating inflammation and the immune response, which are produced in the process of microbial fermentation of indigestible dietary fibers or resistant starch (20, 21). The main SCFAs are acetate, propionate, and butyrate (Figure 1) (22). In the human gut, members of the *Bacteroides phylum* provide most of the acetate and propionate. Butyrate production is mainly by *Firmicutes* (23, 24). An increase in SCFA levels is commonly achieved through a high-fiber diet (25). SCFAs are recognized by receptors on intestinal endocrine cells and intestinal epithelial cells and have anti-inflammatory effects on the intestinal mucosa. SCFAs exert anti-inflammatory effects beyond the gut, which help increase the number of Tregs and inhibit the differentiation of Th17 cells (26, 27) (Figure 1). Tregs can suppress the function of antigen-presenting cells and other effector T cells and suppress immune responses (28). Therefore, Tregs play a significant role in maintaining immune homeostasis and immune tolerance (29). However, Th17 cells are critical for mediating chronic inflammation in autoimmune diseases, producing cytokines such as IL-17, IL-21, IL-22, with pro-inflammatory phenotype (30). It has been shown that *Bacteroides, Enterobacteriaceae, and Sphingobacteriaceae* are positively correlated with some autoimmune diseases, while SCFAs are generally negatively correlated with these diseases (31).

Through protein-coupled receptors [also known as free fatty acid receptors (FFARs)] (Figure 1), signals are transmitted to several non-intestinal cell types (32). One of the receptors, G protein-coupled receptor 109A/ hydroxy carboxylate 2 receptor (GPR109A/HCA2), is activated by butyric acid in the immune system (33). The interaction of butyrate with GPR109A/HCA2 is involved in the development of Tregs (34, 35), which is the basis for regulating homeostasis and maintaining pathogen immune balance and symbiotic bacterial immune tolerance. Depending on the different cytokine environments, the interaction between SCFAs and FFARs affects not only the differentiation of T cells into Tregs but also the differentiation of T cells into effector T cells. Butyric acid regulates gene expression by inhibiting histone deacetylase (HDAC) (Figure 1), especially HDAC1 and HDAC3 (36), which is another mechanism of immune regulation by SCFAs (37). In addition, propionic acid can be used as a weak inhibitor of HDAC (38). Recent studies have shown that inhibition of HDAC may promote the development and function of Tregs, one of the mechanisms by which the gut microbiota enhances intestinal Treg production (39). SCFAs such as butyric acid and propionic acid also inhibit DCs development and regulate antigen presentation by inhibiting HDAC and interacting with FFARs (40–42).

Colonization of the gut with SCFA-producing bacteria also decreases the permeability of the BBB, suggesting that SCFAs play an important role in the development and maintenance of the BBB (27). SCFAs can not only cross the BBB and control neuroimmune homeostasis but also exert a significant role in the recovery and maturation of microglial function (43) (Figure 1). Global defects in the maturation and function of microglial cells have both been found in mice deficient in SCFAs receptors and germ-free mice to display, resulting in the impaired cellular defense that could be rescued by treatment with SCFAs (44).

### 2.3. Immune cells

The gut microbiota and their metabolites influence the activation and differentiation of intestinal B and T cells. Subpopulations of gut T and B cells can circulate from the gut to the meninges, where they release cytokines (45, 46). Cytokines (e.g., IL-10) and antibodies (e.g., IgA) induced by the gut microbiota enter the systemic circulation and influence the local neuroimmune microenvironment through the blood-brain barrier (BBB). These cytokines act on central neurons and microglia to prevent meningeal infection (46). In addition, specific members of the microbiota can also induce defined T cell subsets. *Segmented filamentous bacteria* can induce the development of IL-17A-producing T helper 17 (Th17) cells in the mouse small intestine (47, 48). However, the human gut bacterium *Bacteroides fragilis* improves the development of IL-10-producing Tregs *via* capsular expression of polysaccharide A (PSA) in the mouse colon (49, 50). The gut microbiota also influences microglial maturation and function. Microglia are the most abundant resident immune cells in the brain and are considered to be endogenous macrophages of the CNS, which are essential for tissue homeostasis.

### 2.4. Microbe-associated molecular patterns (MAMPs)

MAMPs (LPS, flagellins, bacterial lipoproteins and unmethylated CpG motifs) recognized by Toll-like receptors (TLRs), and activate immune cells (51). TLRs are expressed by many immune cells, including T cells, B cells macrophages, DCs, and neutrophils, which exert a significant role in molecular communication between changes in the gut microbiota and homeostasis of immune system (52). Cytokine environment is significantly affected by the type and degree of TLRs activation. For example, activation of TLR2 may induce T cells to differentiate to Th2 phenotype through the production of IL-10 and IL-13. However, activation of TLR4 and TLR9 can induce the production of IL-12 through DCs to differentiate T cells to Th1 phenotype (53–55). TLR4-induced IL-23 also contributes to Th17 cells proliferation (56). Once TLRs are activated, pro-inflammatory cytokines IL-1α, I L - 1β, I L - 6, TNF α produced by gut-associated immune cells can cross the BBB to the brain *via* diffusion or cytokine transporters, where they act on receptors on microglia and stimulate further cytokine release from microglia (57).

### 2.5. Neurotransmitters

Levels of host neurotransmitters can be regulated by the gut microbiota, which have an extensive crosstalk with immune cells. Neurotransmitters are actively involved in various brain functions including movement, emotion, learning, and memory (58, 59). Neurotransmitters such as glutamate, acetylcholine (ACh), and dopamine have excitatory effects on neurons GABA, glycine, and serotonin have inhibitory effects on neuron (60, 61). Imbalances of these neurotransmitters can lead to neurological and psychological disorders such as Alzheimer's disease, Parkinson's disease, autism spectrum disorder, anxiety disorders, and depressive disorders (62).

#### 2.5.1. Tryptophan and serotonin

Serotonin, which is also known as 5-hydroxytryptamine (5-HT). Certain resident bacteria, such as *Streptococcus* and *Enterococcus*, produce 5-HT directly (63). *Bifidobacterium infantis* has been demonstrated to affect central 5-HT by elevate plasma tryptophan levels, a precursor of 5-HT (64). 5-HT is involved in the regulation of numerous physiological processes, including respiration, gastrointestinal peristalsis and secretion, vasoconstriction, neurological function, and behavior. Several of the 5-HT receptors are associated with immune cells such as DCs, macrophages, monocytes, and lymphocytes (65). The expression of 5-HT receptors (5-HTR's) on T cells, specifically mRNA expression for 5-HTR_1a_ and 5-HTR_2_ in naive CD4 T cells and Th1, Th17, and Tregs (66, 67). Indeed, 5-HT has been shown to activate T cells *via* 5-HTR_3_-mediated signaling, increase intracellular Na^+^ and facilitate T cells proliferation (68). These open up new avenues of understanding how 5-HT influences T cells function.

#### 2.5.2. GABA

As well as producing precursors, many bacteria can synthesize and release neurotransmitters, several microorganisms such as *Bacteroides, Parabacteroides*, and *Bifidobacterium Escherichia* produce GABA (69). In addition, *Lactobacillus rhamnosus* produces GABA and regulates GABAAα2 and GABAB1b receptors in the brain, thereby attenuating anxiety-like and depression behavior in mice (13). In the immune system, the GABAergic signaling system plays a key role in response to various inflammatory disorders and influences various functional properties of the immune cells, such as LPS-induced cytokine release, antigen-induced T-cells proliferation, cytotoxicity and chemotaxis of effector T-cells activity (70). GABA transporters that critically implicated in GABA secretion have been identified in mononuclear macrophages and CD4 T cells (71). GABA transporter GAT-1 is expressed on activated T cells, and inhibits the proliferation of CD4 T cells (72). GABA acts as a negative regulator of macrophage and microglial production of inflammatory cytokines and T cell activation *via* regulating the function of antigen presenting cells (APC) (71, 73) and blocking calcium signaling and NF-κ activity (74).

#### 2.5.3. Dopamine

Dopamine, a precursor for other catecholamines, like epinephrine (E) and norepinephrine (NE), associated with a variety of neurological processes, including motor control, cognition, learning and reward, which can be produced by *Bacillus* (75). Dopamine stimulates T cells activation resulting in TNFα and IL-10 cytokine production from naive T cells (76). By activating T cell D1 receptors, dopamine activates T cells *via* inhibiting function of Tregs (77). NE is known for its role in behavior and cognition, like memory, learning, and attention, which can be produced by *Saccharomyces, Escheridia, and Bacillu s*(78). By binding to adrenergic receptors, E and NE regulate immune activity (79). NE restricts the activation of microglia and diminishes pro-inflammatory mediators production including IL-1β, TNF-α (80).

#### 2.5.4. Ach

ACh, synthesized and released by the parasympathetic nerves, directly affects immune cells *via* muscarinic Ach receptors and nicotinic Ach receptors, which can also be produced by *Lactobacillus*(81, 82). ACh exerts an anti-inflammatory effect on macrophages, mast cells, and basophils *via* α7-nicotinic acetylcholine receptors (α7nAChRs) (83), receptors on inflammatory macrophages. Activation of the cholinergic system by α7nAChR reduces inflammation levels in experimental autoimmune encephalomyelitis (EAE) (84). Also, ACh treatment may diminish the levels of pro-inflammatory cytokines in the blood such as IL-1β, IL-6, and TNF-α in mice (85). Furthermore, ACh implicates in the anti-inflammation *via* down-regulation inflammatory cytokine synthesis and inhibition of NF-κB nuclear translocation (86). Since acetylcholinesterase (AChE) inactivates acetylcholine, Li et al. reported that inhibition of AChE can play a positive role in the treatment of autoimmune diseases (87).

### 2.6. HPA axis

Neurotransmitters and cytokines coming from the above systems might influence the. Within the nervous system, stress can also activate the HPA axis response (88–90). Activation of HPA axis causes hypothalamic neurons to secrete corticotropin receptor hormone (CRH), which enters the brain or portal circulation, triggering the release of corticotropin (ACTH), which then initiates the synthesis and release of glucocorticoids (GCs). As a mediator between the nervous and immune systems, GCs has an inhibitory effect on almost all immune cells, including lymphocytes, macrophages, neutrophils, mast cells and so on. During acute stress, through the HPA axis, adrenal corticokinin concentration in blood can be increased and immune function can be inhibited, which is one of the main ways for stress to inhibit immune function (91, 92). They may also promote the development of pathogenic Th cells and cause tissue damage through neural and intestinal inflammation, in turn, affect intestinal barrier integrity (93).

In conclusion, the gut microbiota regulates brain development by the bidirectional pathway of the GBA; thus, it can change host behavior and affect the occurrence and development of diseases (94). Studying the roles and mechanisms of microorganisms associated with the GBA will provide new means for diagnosing and treating neuroimmune diseases.

## 3. Fecal microbiota transplantation (FMT)

FMT refers to transplanting the gut microbiota from a healthy donor's feces into a patient's gastrointestinal tract to rebuild intestinal microecology of the recipient (95). Recent evidence suggests that the gut microbiota probably plays a causal role in the pathophysiology and pathogenesis of some diseases. Therefore, changing the composition of gut bacteria may affect the course of some diseases. In most cases, the causal pathogen is unlikely to be a single microorganism. FMT can remedy an altered structure of gut microbiota in disease to a certain degree. Therefore, compared with probiotics, one superiority of FMT is the transplantation of intact and healthy gut microbiota. FMT has been successfully used in recurrent or refractory Clostridium infection. FMT is widely used because of its high safety and good efficacy and plays a crucial role in the treatment of autoimmune enteritis, hepatitis B, chronic nonalcoholic cirrhosis, epilepsy, and autism spectrum disorder (96). However, as a biological treatment, FMT also has side effects similar to all medicines and medical therapies. The most common side effects after FMT are often related to constipation, diarrhea, abdominal pain, and transient low-grade fevers, all of which may subside within a few days to weeks (97–99). These side effects are the natural response of the body after the introduction of live microbes and their metabolites. Although extremely rare, serious adverse events and mortality have been reported, but in many instances, have been found as a result of comorbidities or causes other than FMT.

## 4. The gut microbiota and FMT in neuroimmune diseases

Emerging data have suggested that gut microbiota dysbiosis may be related to the progress of neuroimmune diseases, namely, myasthenia gravis (MG), multiple sclerosis (MS), neuromyelitis optica spectrum disorders (NMOSDs), autoimmune encephalitis (AIE), and Guillain–Barré syndrome (GBS). The exact pathogenesis of these diseases is not completely clear. FMT, which is aimed at reconstructing the gut microbiota, has been proposed as a new therapy for neuroimmune disease treatment. In addition to the gut microbiota, bacterial metabolites (SCFAs) are associated with these diseases. The roles of the gut microbiota and the effects of FMT in neuroimmune diseases are summarized below.

### 4.1. Myasthenia gravis (MG)

MG is an autoimmune disease caused by autoantibodies that target the neuromuscular junction, resulting in muscle weakness and fatigue (100). Patients with MG whose weakness is confined to the extraocular muscles are referred to oculomotor myasthenia gravis (OMG). Ocular muscle weakness is the most common presenting symptom. Once fatigue spreads to the bulbar, limb, axial, and ventilator muscles, the disorder can progress to generalized myasthenia gravis (GMG) (101). The typical pathological change in MG patients is the formation of germinal centers in the thymus (102). Acetylcholine receptor (AChR) antibodies can be detected with routine assays in 70–80% of MG patients, and antibody titers tend to correlate with disease severity. The production of AChR antibodies is relevant to the disequilibrium of Th17 and Treg cells (103). Hence, rebuilding the Thl7/Treg balance has promising applications for the biological target therapy of MG.

Studies on the close relationship between severe MG and intestinal microbiota have found lower microbial diversity in MG patients than in HCs, suggesting an abnormal microbial status (104, 105). In particular, SCFAs in the feces of MG patients are significantly reduced compared with those in the feces of HCs. Moreover, phylum-level analyses have indicated the decreased abundance of the phylum Firmicutes along with an increased abundance of the phyla *Bacteroidetes and Proteobacteria* in MG patients' fecal samples. Hence, the ratio of *Firmicutes/Bacteroidetes* (F/B ratio) is obviously low in MG patients (106), which would reduce the production of SCFAs, resulting in modifications of immune homeostasis and intestinal permeability, thus leading to the exacerbation of autoimmune diseases (107). In another study, compared with the HCs, the MG patients were found to harbor significantly lower relative proportions of the families *Bifidobacteriaceae* and *Verrucomicrobiaceae* as well as *Coriobacteriaceae, Flavobacteriaceae*, and *Leuconostocacceae*. In contrast, the MG patients were shown to harbor higher proportions of *Desulfovibrionaceae, Acidaminococcaceae*, and *Pasteurellaceae*. At the genus level, the relative abundances of *Clostridium* and *Eubacterium* were sharply lower in the MG patients, while the proportions of *Streptococcus* and *Parasutterella* were enriched (108). Surana et al. found that the main characteristic of MG patients was a decrease in OTUs belonging to *Lachnospiraceae* (23 OTUs) and *Ruminococcaceae* (8 OTUs) (109) (Table 1). *Lachnospira* and *Ruminococcaceae* are two of the most abundant families in *Clostridiales* and have been associated with the maintenance of gut health. There is ample evidence demonstrating that the abundance of clostridia has profound effects on the production of SCFAs (110). These SCFAs could affect T cells by regulating their differentiation into Tregs. To directly examine the effect of Clostridium on the induction of colonic Tregs, Atarashi et al. used chloroform to treat the feces of conventionally reared mice. Then, they obtained 46 strains of clostridia and inoculated them into GF mice, inducing a robust accumulation of Tregs in the colons lamina propria (LP) of these mice (125). It provides new insights into the treatment of MG at the microbial level.

**Table 1:** The alterations of gut microbiota in neuroimmune diseases.

**Disease**	**Species**	**Alterations of gut microbiota**	**After FMT**	**Reference**
Myasthenia gravis	Human	Decreased: *Firmicutes, Bifidobacteriaceae, Verrucomicrobiaceae, Coriobacteriaceae, Flavobacteriaceae, Leuconostocacceae, Clostridium, Eubacterium, Lachnospiraceae, Ruminococcaceae*		(106, 108, 110)
		Increased: *Desulfovibrionaceae, Acidaminococcaceae, Pasteurellaceae Proteobacteria, Bacteroidetes, Tricspiraceae*		
	Mice	Decreased: *Rumataceae*		(111)
Multiple sclerosis	Human	Decreased: *Lachnospiraceae, Bacteroides, Faecalibacterium, Prevotella, Butyricimonas, Paraprevotella, Haemophilus, Slackia, Anaerostipes, Adlercreutzia*		(112–117)
		Increased: *Bifidobacterium, Streptococcus, Methanobrevibacter, Akkermansia*		
	Mice	Decreased: *Bacteroid, Lactobacillus*	Decreased: *Bacteroides, Firmicutes, Tenericutes Actinobacteria, Bacteroides, Firmicutes, Tenericutes Actinobacteria, Cyanobacteria*	(118, 119)
		Increased: *Streptococcus, Firmicutes, Tenericutes, Cyanobacteria*	Increased: *Proteobacteria*	
Neuromyelitis optica spectrum disorders	Human	Decreased: *Clostridium, Parabacteroides, Oxalobacter, Burkholderia*		(120, 121)
		Increased: *Streptococcus, Alistipes, Haemophilus, Veillonella, Butyricimonas, Rothia*		
Autoimmune encephalitis	Human	Decreased: *Faecalibacterium, Roseburia, Lachnospira, Ruminococcus, Coprococcus, Dialister, Collinsella, Anaerostipes*		(122)
		Increased*: Bacteroides, Enterococcus, Escherichia, Veillonella, Streptococcus, Dorea, Scardovia, Clostridium*		
Guillain-Barré syndrome	Human	Increased: *Campylobacter jejuni*		(123, 124)

Experimental autoimmune myasthenia gravis (EAMG) in the susceptible Lewis rat is a well-accepted animal model elucidating the pathogenesis of the disease and developing new or improved MG therapies. Rinaldi et al. found a strong reduction in *Lachnospiraceae* abundance in chronic EAMG mice (Table 1) and an increase in the *Ruminococcaceae/Lachnospiraceae* (R/L) ratio (111). In a study, the total distance traveled by GF mice colonized with MG microbiota (MMb) was substantially decreased relative to the mice colonized with healthy microbiota (HMb) during the open-field test (OFT). However, this effect could be reversed after co-inoculation with both MMb and HMb, indicating that an intervention with the microbiota may be a potential therapeutic strategy for MG (31).

### 4.2. Multiple sclerosis (MS)

MS is an autoimmune disease characterized by progressive demyelination and deterioration of neurologic function (126, 127). There are four clinical forms of MS: primary progressive MS (PPMS), relapsing-remitting MS (RRMS), secondary progressive MS (SPMS), and progressive relapsing MS (PRMS) (128, 129). Among them, the most common phenotype of MS (80% of cases) is RRMS, characterized by clearly defined attacks or relapses, followed by a variable degree of recovery (130).

Recently, the role of gut microbiota in the development of multiple sclerosis has received increasing attention, as well as SCFAs. In a Chinese cohort study, fecal SCFAs were discovered to be reduced in MS compared to HCs (131).The majority of intestinal dysbiotic microbiota data for patients with MS are characterized by lower abundances of *Butyricimonas*, a butyrate-producing genus, in MS patients. These changes may increase the pro-inflammatory autoreactive T cells, i.e., Th17 and Th1 cells in peripheral blood. Increased IL-17 mRNA was first noted in the blood and CSF of MS patients (112). Subsequently, increased Th17 cells and IL-17 protein were found in the brains of MS patients (132). This leads to increased BBB permeability, which in turn leads to increased CNS inflammation (133). Altered intestinal microbiota communities are related to autoimmune responses and systemic inflammatory in the host (117). Indeed, concentrations of fecal SCFAs have been shown to be decreased in RRMS patients compared to HCs (134). Hence, inflammatory trigger could be mediate by a low SCFA-producing microbial community in MS patients (135). Butyrate, produced by *Faecalibacterium, Lachnospiraceae*, and *Anaerostipes*, inhibits to suppress CNS demyelination *via* G-protein-coupled receptor activation and histone deacetylase (113), the main pathological feature in MS. Butyrate can also enhance barrier function and anti-inflammatory activities (26, 136, 137). Longitudinal data for 97 patients with MS who underwent propionate supplementation for at least 1 year showed a reduced annual relapse rate, disability stabilization, and reduced brain atrophy (116, 138). Hence, SCFA supplementation is efficacious in reducing MS clinical severity and inflammation.

Compared with HCs, MS patients have been found to harbor significantly lower relative proportions of *Bacteroides, Faecalibacterium, Prevotella, Butyricimonas, Paraprevotella, Haemophilus, Slackia*, and *Anaerostipes*. In contrast, MS patients have been shown to harbor higher proportions of *Bifidobacterium, Streptococcus, Methanobrevibacter*, and *Akkermansia* (114, 115). A lower abundance of the phytoestrogen-metabolizing bacterium *Prevotella, Parabacteroides*, and *Adlercreutzia* were observed in RRMS patients than in HCs (116, 117) (Table 1). Treatment with estrogens can suppress MS patients' symptoms (139). In a patient with SPMS complicated with recurrent Clostridium difficile infection (CDI), FMT delayed the disease progression and alleviated the recurrent infection of MS, but the Expanded Disability Status Scale (EDSS) score of the patient was stable, and the symptoms did not improve. After FMT treatment for severe constipation, three wheelchair-bound MS patients could defecate normally, and they had a dramatic improvement in neurological symptoms and were able to walk unassisted. Therefore, although the therapeutic effect of FMT is limited, it appears to provide long-term benefits for patients with MS (140).

By using an EAE mouse model, which can best simulate the clinical manifestations and pathophysiological characteristics of MS. Wang et al. found that *Adlercreutzia* was the most abundant genus related to the differentially expressed genes (DEGs) of the spinal cord in EAE mice treated with FMT. However, the DEGs associated with inflammation were negatively correlated with the relative abundance of *Adlercreutzia*. These results indicate a potential *Adlercreutzia*-mediated immune regulation mechanism for FMT treatment of EAE (118). Compared with HCs, EAE mice exhibited significantly decreased relative abundances of total gut microbiota, *Bacteroides*, and *Lactobacillus* and considerably increased abundances of *Streptococcus, Firmicutes, Tenericutes*, and *Cyanobacteria* (119). Oral administration of the fecal microbiota of MS patients can aggravate the symptoms of EAE mice and reduce the level of the anti-inflammatory cytokine IL-10. FMT-treated EAE mice present changes in abundances of *Verrucomicrobia* and six intestinal bacterial phyla, *Bacteroidetes, Firmicutes, Tenericutes, Cyanobacteria, Proteobacteria*, and *Actinobacteria*, all of which were converted to normal control levels. In addition, FMT can reduce the abundance of *Bacteroides* and *Actinobacteria* (118) (Table 1). These results suggest that FMT can alter the structure of gut microbiota in EAE to a certain extent. Compared with those of EAE controls, FMT treatment not only alleviated clinical symptoms, but also significantly reduced clinical scores and cumulative disease scores in mice throughout the clinical course of EAE (141). Li et al. also found that FMT of healthy mice alleviates the symptoms of EAE mice by restoring the integrity of the BBB and axon myelination (142).

### 4.3. Neuromyelitis optica spectrum disorders (NMOSDs)

NMOSDs are disabling, sometimes fatal CNS inflammatory demyelinating diseases encompassing a brain syndrome, optic neuritis, and acute myelitis (143). Although traditionally considered a severe atypical form of MS, NMOSD is now recognized as a distinct clinical entity (144). Compared with HCs, patients with NMOSD have more aquaporin (AQP) autoantibodies against the optic nerve and spinal cord (145).

A striking depletion of fecal SCFAs with a significantly negative correlation with disease severity has been observed in NMOSD patients (146), showing that the significant reduction in fecal SCFA levels may become another important feature of NMOSD patients. Correlation analysis shows significant reductions in fecal butyrate levels in NMOSD patients. SCFAs play a critical regulatory role in host physiology and immunity. SCFAs have anti-inflammatory effects that are not limited to the intestinal tract, which help increase Treg and suppress the differentiation of Th17 cells. Increased Th17 cells and IL-17 have been noted in patients with NMOSD (147). Therefore, the lack of protection from anti-inflammatory metabolites of beneficial bacteria is also involved in the pathogenesis of NMOSD (146).

The gut microbial composition of NMOSD patients is distinguished from that of HCs. Generally speaking, the gut microbiota of NMOSD patients has a high abundance of *Streptococcus, Alistipes, Haemophilus, Veillonella, Butyricimonas*, and *Rothia*. However, the abundance of *Clostridium, Parabacteroides, Oxalobacter*, and *Burkholderia* abundances is low (120) (Table 1). *Streptococcus*, which is significantly increased in NMOSD patients, is positively correlated with disease severity. The use of immunosuppressants results in a decrease in *Streptococcus*, suggesting that *Streptococcus* might exert a significant role in the pathogenesis of NMOSD. Remarkably, of bacteria identified at the species level, *C. perfringens* was the species most significantly enriched in patients with NMOSD compared with HCs (121).

### 4.4. Autoimmune encephalitis (AIE)

AIE is a neurological disorder caused by inflammation of the brain parenchyma. Its most common cause is an underlying viral infection, but autoimmune factors are increasingly being considered the cause of encephalitis (148, 149). Different antibodies directed mainly toward synaptic receptors, including the N-methyl-D-aspartate receptor (NMDAR), the α-amino-3-hydroxy-5-methyl-4-isoxazole-propionic acid receptor (AMPAR), and the GABA B-receptor (GABABR), contribute to the development of AIE. It has been reported that anti-NMDAR encephalitis is the most prevalent and severe autoimmune encephalitis type (150).

Relative to HCs, anti-NMDAR encephalitis patients have a decreased microbiome alpha-diversity index and marked disturbances in the gut microbial composition. Chen et al. observed a decrease in various SCFA-producing bacteria such as *Faecalibacterium, Roseburia, Lachnospira, Ruminococcus, Coprococcus*, and *Collinsella* and an increase in *Bacteroides, Enterococcus, Escherichia, Veillonella, Streptococcus, Dorea, Scardovia* and *Clostridium* in anti-NMDAR encephalitis patients relative to HCs (122) (Table 1). The reduction in SCFA production by the microbiome increases the destruction of the intestinal barrier (151). In verifying intestinal mucosal damage, they found that patients with anti-NMDAR encephalitis had significantly elevated serum levels of two chemical markers, D-LAC and DAO, which are usually at higher levels during intestinal barrier disruption (152). Notably, *Streptococcus*, the tax on most closely associated with NMOSDs, has also been shown to be associated with anti-NMDAR encephalitis and D-LAC, suggesting that it is involved in intestinal mucosal damage (146). Therefore, a lack of protection from anti-inflammatory metabolites produced by beneficial bacteria and abnormal intestinal permeability also take part in the pathogenesis of anti-NMDAR encephalitis. Related to HMb mice, microbiota-depleted mice receiving FMT from anti-NMDAR encephalitis patients had cognitive impairment and hypersensitivity. Furthermore, *via* FMT, transplantation of anti-NMDAR encephalitis patients into specific pathogen-free (SPF) mice can induce Th17 response and abnormal behaviors. These results suggest the potentially important involvement of the gut microbiota in anti-NMDAR encephalitis (122).

### 4.5. Guillain–Barré syndrome (GBS)

GBS is a paralytic neuropathy and is considered to be predominantly preceded by an autoimmune response after gastrointestinal infection with *Campylobacter jejuni* (Table 1) or other immune stimulation. GBS is frequently characterized by the rapid progression of bilateral weakness, combined with autonomic or additional neurologic symptoms. The majority of patients improve, however, permanent disability can occur in some patients (153). The innate immune response to campylobacteriosis is manifested by the accumulation of neutrophils and macrophages, inflammatory mucosal damage, intestinal barrier defects, malabsorption, and ultimately bloody diarrhea (154).

Mice inoculated with *Campylobacter jejuni* from GBS patients show increased levels of autoantibodies and peripheral nerve injury (123, 124), suggesting that intestinal microbiome dysregulation is closely related to the pathogenesis of GBS. The cross-reaction between LPS on the surface of *Campylobacter jejuni* and autoimmune anti-ganglioside antibody resulted in complement-mediated nerve damage (155). In addition, Brooks et al. found that Th2 and autoimmune responses in mice infected with *Campylobacter jejuni* increased after treatment with human FMT. The combination of FMT and antibiotics obviously accelerated the clearance of *Campylobacter jejuni* in infected mice (156).

## 5. Conclusion

The gut microbiota regulates the function of the brain by altering the microbiota composition, diversity, and metabolites through the bidirectional pathways between the gut and brain; thus, it can alter host behavior and affect the progression of neuroimmune diseases. FMT positively impacts the composition and function of microbiota by increasing the level of SCFAs. Hence, it is theoretically feasible to apply FMT to diseases with Th17/Treg cell imbalances. Therefore, FMT may be an effective therapy for treating human neuroimmune diseases. However, the evidence level of current clinical research is not sufficient. To date, studies on FMT for many neuroimmune diseases have been limited to animal models. Notably, whether the results obtained in animal experiments can be directly applied to human patients is unclear. At present, further experimental and clinical studies are needed to clarify the mechanism of the gut microbiota and its metabolites in neuroimmune diseases. Nevertheless, transplantations of anti-inflammatory strains and SCFAs seem to be helpful in the treatment of neuroimmune diseases and will provide more methods for treating neuroimmune diseases.

## Author contributions

All authors listed have made a substantial, direct, and intellectual contribution to the work and approved it for publication.
